# The Digital Brain Tumour Atlas, an open histopathology resource

**DOI:** 10.1038/s41597-022-01157-0

**Published:** 2022-02-15

**Authors:** Thomas Roetzer-Pejrimovsky, Anna-Christina Moser, Baran Atli, Clemens Christian Vogel, Petra A. Mercea, Romana Prihoda, Ellen Gelpi, Christine Haberler, Romana Höftberger, Johannes A. Hainfellner, Bernhard Baumann, Georg Langs, Adelheid Woehrer

**Affiliations:** 1 Division of Neuropathology and Neurochemistry, Department of Neurology, Medical University of Vienna, Vienna, Austria; 2 Department of Neurosurgery, Medical University of Vienna, Vienna, Austria; 3 Department of Neurosurgery, University Hospital St. Poelten, St. Poelten, Austria; 4 Center for Medical Physics and Biomedical Engineering, Medical University of Vienna, Vienna, Austria; 5 Department of Biomedical Imaging and Image-Guided Therapy, Computational Imaging Research Lab, Medical University of Vienna, Vienna, Austria

**Keywords:** CNS cancer, Cancer in the nervous system, Cancer microenvironment, CNS cancer

## Abstract

Currently, approximately 150 different brain tumour types are defined by the WHO. Recent endeavours to exploit machine learning and deep learning methods for supporting more precise diagnostics based on the histological tumour appearance have been hampered by the relative paucity of accessible digital histopathological datasets. While freely available datasets are relatively common in many medical specialties such as radiology and genomic medicine, there is still an unmet need regarding histopathological data. Thus, we digitized a significant portion of a large dedicated brain tumour bank based at the Division of Neuropathology and Neurochemistry of the Medical University of Vienna, covering brain tumour cases from 1995–2019. A total of 3,115 slides of 126 brain tumour types (including 47 control tissue slides) have been scanned. Additionally, complementary clinical annotations have been collected for each case. In the present manuscript, we thoroughly discuss this unique dataset and make it publicly available for potential use cases in machine learning and digital image analysis, teaching and as a reference for external validation.

## Abstract (summary)

**Table:** 

Measurement(s)	Cancer Histology • Cellularity Measurement • Total Sample Tissue Area • brain neoplasm
Technology Type(s)	Hematoxylin and Eosin Staining Method • digital curation • Histology and Immunohistochemistry Shared Resource
Factor Type(s)	age • sex • location
Sample Characteristic - Organism	Homo sapiens

Machine-accessible metadata file describing the reported data: 10.6084/m9.figshare.16652272

## Background & Summary

Brain tumours account for a large fraction of years of potential life lost as compared with tumours from other sites^1^, and have a significant negative impact on patients’ quality of life^2^. Overall, they are relatively uncommon neoplasms with an incidence of approximately 24 per 100.000 person-years^3^. Current diagnostic guidelines published by the WHO define approximately 150 distinct brain tumour types and assign grades I to IV, based on malignancy and potential to malignant transformation or progression. They are mainly differentiated by their histopathological phenotypes and molecular alterations^4^. While the majority of tumours is diagnosed solely based on histopathology, an integrated approach is mandatory for 19 tumour types.

Still, more accurate diagnostic distinctions are needed in order to i) better assess individual patients’ prognoses and ii) support more robust therapeutic decisions^4,5^. Recently, diagnostic algorithms trained on DNA methylation data have been shown to significantly increase diagnostic accuracy^6^. Similar advances focusing on histopathological data have been hampered, so far, by the lack of freely available histopathology datasets^7^. Most available histopathology data such as those available through TCGA^8^, IvyGAP^9,10^ or TCIA^11^ focus on only a few diagnostic entities. They mostly consist of digitized fresh frozen tissue sections, which feature relatively poor tissue morphology as compared to formalin-fixed and paraffin-embedded tissues. Still, even with these limited data, computational algorithms have been successfully trained - amongst others - for survival prediction^12^, detection of tumour-infiltrating lymphocytes^13^, and assessments of tumour microvessels^14^. However, larger datasets encompassing an even wider range of brain tumours and featuring improved cellular and morphological characteristics are necessary to further develop these algorithms and extend their applicability to the entire spectrum of brain tumour types.

**Fig. 1:**
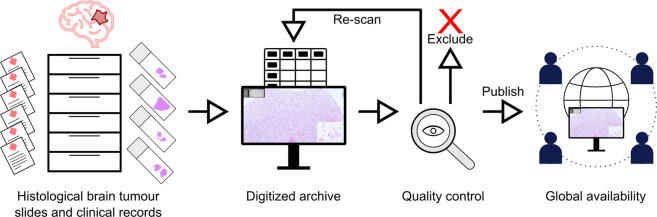
Overview of the data acquisition and publication process. First, histological slides and clinical records of brain tumour patients were retrieved from the biobank of the Division of Neuropathology and Neurochemistry, Medical University of Vienna. Then, slides were digitized using a Hamamatsu slidescanner. Clinical data were translated into standardized annotations. At least two experienced neuropathologists checked each slide scan to ensure conformity of the diagnosis with the current revised 4th edition of the “WHO Classification of Tumours of the Central Nervous System” and sufficient scan quality. Ambiguous cases were excluded and WSIs of inferior quality were re-scanned. Finally, data were made available via EBRAINS to the international research community. (Brain illustration adapted from Meaghan Hendricks from the Noun Project).

Thus, we set out to compile a comprehensive resource of digitized Haematoxylin-eosin(H&E)-stained brain tumour whole slide images (WSIs) with clinical annotations (Fig. 1). We aimed to capture the complete spectrum of brain tumours as encountered in day-to-day medical diagnostic practice. Importantly, we managed to specifically digitize slides of exceedingly rare pathologies, which are usually, if ever, seen only a few times in a pathologist’s lifetime. By performing a manual review of each slide, we ensure high scan quality and actuality of provided diagnoses. We envisage this dataset to be used for advancing digital pathology-based machine learning and for teaching purposes. Importantly, this dataset can be used for (1) inter-tumour comparisons thanks to the wide inclusion of distinct brain tumour types as well as (2) within-tumour-type investigations thanks to the inclusion of a large number of samples for the common tumour types.

## Methods

### Sample acquisition

H&E stained tumour slides from FFPE tissues, which were collected for routine diagnostics in the time interval of 1995–2019 have been obtained from the biobank of the Division of Neuropathology & Neurochemistry, Medical University of Vienna. We digitized each slide in high magnification (40x objective, 228 nm/pixel) using a Hamamatsu NanoZoomer 2.0 HT slide-scanner. Each slide was manually reviewed to ensure high scan quality and sufficient diagnostic tumour tissue. Samples with equivocal diagnoses or missing molecular work-up otherwise needed to assign an integrated WHO 2016 diagnosis were excluded. A subset of glioblastoma scans (n = 381) has been published previously as part of the GBMatch study^15^.

Basic clinical annotations consisting of patient age and sex as well as tumour location and recurrence were acquired from local electronic records where available. Tumour locations have been assigned to the following 19 categories: frontal; parietal; insular; occipital; temporal; cerebellar; brain stem; spinal; lateral ventricle; diencephalon; third ventricle; fourth ventricle; sellar region; cranial nerves; basal ganglia; cerebral, NOS (not otherwise specified); posterior fossa, NOS; cranial, NOS; and other.

This study complies with the relevant ethical, legal and institutional regulations and the study protocol has been approved by the Ethics Committee of the Medical University of Vienna (EK1691–2017). Participant informed consent has been obtained as by institutional guidelines, necessitating restrictions on commercial use of the obtained data.

### Estimation of cell density and scanned tissue area

Additionally, the total tissue area and the average cellularities were estimated for each scan using a custom MATLAB script (MATLAB R2017b, MathWorks) with a similar approach as previously published^15,16^. In summary, H&E stained WSIs were first colour-deconvoluted into separate Haematoxylin and Eosin channels^17^. Then, global, Phansalkar and Otsu thresholding were applied to the Haematoxylin channel to identify nuclei^18,19^. Watershedding was used to separate densely clustered cells^20^. Only cells with a minimum size of 4 pixels were kept. The total tissue area was determined by averaging all colour channels, thresholding at a threshold of 220, followed by binary *close* and *open* operations.

## Data Records

Data are provided via EBRAINS^21^ as one ndpi-file per sample, sorted by diagnostic tumour type (in alphabetical order) for easier access. It is possible to download single files directly or all files of a specific tumour type or the whole dataset using a download manager (such as the Chrono Download Manager for the Google Chrome browser). Furthermore, supplementary clinical information, estimated cell densities and scanned tissue area is provided in a csv-spreadsheet with one row per tumour sample. An overview of all spreadsheet variables and descriptions is given in Table 1.

**Table 1:** Recorded clinical variables and corresponding descriptions.

Variable	Description
**uuid**	unique sample identifier
**pat_id**	unique patient identifier
**diagnosis**	primary diagnosis according to the WHO *Classification of Tumours of the Central Nervous System (2016)*
**grade**	WHO grade according to the WHO *Classification of Tumours of the Central Nervous System (2016)*
**subtype**	further specification of the histopathological subtype which is not a distinct entity as defined by the WHO, if applicable
**secondary_diagnosis**	secondary diagnosis in cases where two distinct diagnosis according to the WHO are applicable
**control**	1 if sample is a control sample without tumour tissue
**age**	patient age at the time of surgery
**sex**	biological patient sex
**location**	list (in square brackets) of all applicable tumour locations; empty if location is unknown
**laterality**	laterality of the tumour (left or right)
**cellularity**	estimated cell density of the tissue (given in 1/mm^2^)
**tissue_area**	estimated scanned tissue area (in mm^2^)
**recurrence**	0 if the entry corresponds to a primary tumour resection; if the entry corresponds to a tumour recurrence, the number of the recurrence is given (e.g., 2 corresponds to the second recurrence)
**comment**	notable findings that do not fit in other columns (e.g., important mutations not yet integrated in the WHO classification; other non-tumour pathologies in the control samples)

**Fig. 2:**
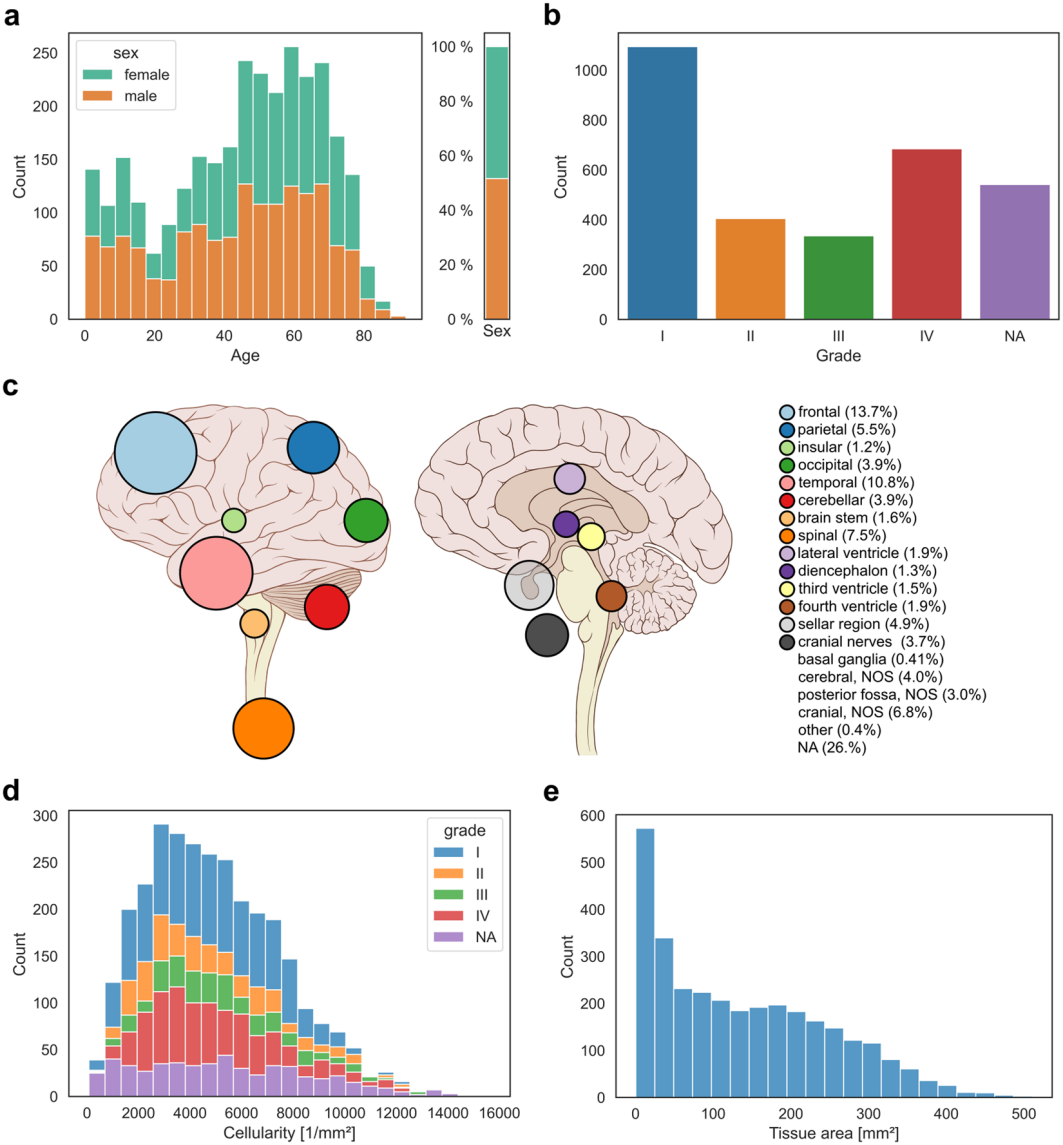
Descriptive statistics of the ‘Digital Brain Tumour Atlas’ patient cohort (not including control patients). (**a**) The age distribution by sex shows a bimodal distribution with most patients belonging to the higher-age categories. Since some uncommon tumour types like medulloblastoma occur mainly in children and have been strategically over-sampled, there is also a peak in younger patients. (**b**) The distribution of the different WHO grades shows a slight predominance of grade I and grade IV tumours. Of note, some tumour entities are not assigned WHO grades (‘NA’) and very few tumour types are assigned intermediate grades II-III (a total of five cases, not shown in the figure). (**c**) Tumour distribution with colour-coded locations and ratio-specific circle sizes. (Brain illustration adapted from Patrick J. Lynch, wikimedia) (**d**) Distribution of the cell densities of all included tumour samples by tumour grade. Note that lower-grade tumours are not necessarily less cell dense (e.g., in the case of cellular schwannoma). (**e**) The distribution of the scanned tissue areas (per slide).

**Fig. 3:**
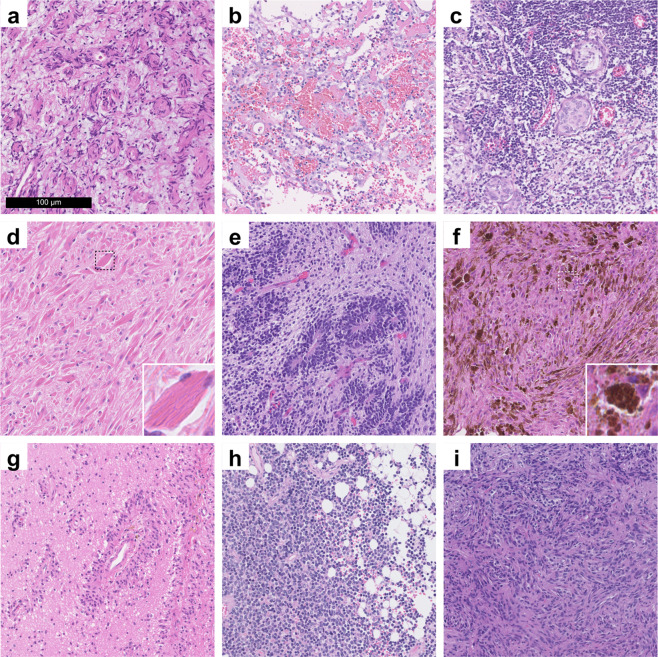
Exemplary images from exceedingly rare brain tumours, which are included in the DBTA. (**a**) Perineurioma component of a hybrid nerve sheath tumour. (**b**) Angiosarcoma. (**c**) Lymphoplasmacyte-rich meningioma. (**d**) Crystal-storing histiocytosis. (**e**) Embryonal tumour with multilayered rosettes. (**f**) Melanotic schwannoma. (**g**) Angiocentric glioma. (**h**) Cerebellar liponeurocytoma. (**i**) Pituicytoma.

A total of 3,115 histological slides of 2,880 patients have been scanned. A total of 126 distinct diagnostic tumour types could be included. There are 1,395 female and 1,462 male patients in the dataset. The mean patient age at brain tumour surgery was 45 years, ranging from 9 days to 92 years. 2,530 of the scanned slides originated from primary operations and 538 from re-operations. See online-only Table 1 for descriptive properties broken down by tumour type. Descriptive visualizations of patient age, sex, tumour location, cellularity, and scanned tissue area are given in Fig. 2. Of note, we also scanned exceptionally rare tumour types such as melanotic schwannomas or liponeurocytomas (Fig. 3). A total of 47 non-tumour slides from different non-tumour CNS regions and with different pathologies were included as controls.

## Technical Validation

All cases were initially selected based on the given diagnosis in the diagnostic electronic records. To ensure conformity with the WHO 2016 diagnosis, all slides have been independently reviewed by two neuropathologists experienced in neuro-oncology. In disputed cases, a third senior neuropathologist was consulted. Older cases with missing necessary molecular analyses were not included in the dataset.

Inter- and intraobserver variability is one factor that contributes to misdiagnoses or discrepant diagnoses. We mitigated the risk by including only cases that had already undergone thorough routine diagnostic work-up and were additionally reviewed independently by at least two neuropathologists as described above. In this way, we also ensured excellent image quality and the presence of sufficient diagnostic tumour tissue on each WSI. Scans with suboptimal image quality were either re-scanned (if possible) or excluded.

## Usage Notes

### Data access

The data can be accessed via EBRAINS^21^. In order to download the data set, users have to register with EBRAINS and agree to the general terms of use, access policy as well as the data use agreement for pseudonymised human data (https://ebrains.eu/terms). The data are distributed under the conditions that users cite the respective DOI, adhere to EBRAINS’ Data Use Agreement and do not use the data for commercial purposes.

### WSI processing

The ndp.view2 (© Hamamatsu) software can be freely used to view and annotate slide scans saved in the ndpi format^22^. Alternatively, most other WSI programs such as the open-source OMERO software platform^23^ and the open-source QuPath software^24^ can work directly on ndpi-files. However, most programming languages and non-specialized image processing software cannot handle ndpi-files out of the box. Thus, we also provide a toolbox of MATLAB scripts that depend on the openslide library^25^ and can be used to

1. Automatically tile large slide scans and export multiple smaller image patches in a given magnification.
2. Convert annotation-files (.ndpa) to overlays, which can be used to extract specific regions of interest.
3. Estimate the total tissue area on a WSI.
4. Estimate the cell density on a WSI.

Of note, slide thickness and staining intensity vary to some degree, resulting in a slightly different histological appearance of each slide. Thus, for machine learning applications, we recommend astain normalization step such as WSICS^26^, more recent methods employing generative adversarial networks^27^ or style transfer learning^28^. Moreover, heavy stain colour augmentation should be performed^29^. Of note, the stain normalization step can be omitted with only a negligible drop in performance as has been shown by Tellez *et al*.^29^.

## Data Availability

The custom-made MATLAB toolbox for loading, viewing and processing of ndpi & ndpa files and for estimating the total tissue area and average cell density of a WSI can be accessed at: https://github.com/tovaroe/WSI_histology.

## Online-only Table

**Online-only Table 1:** Overview of the frequencies and descriptive statistics of all brain tumour types included in the DBTA.

	# of scans	Median age	Age range	Cellularity	Tissue Area	F:M ratio	Most frequent location
Adamantinomatous craniopharyngioma	85	30 years	2–79 years	5219 ± 254/mm^2^	81 ± 7 mm^2^	1.36	sellar region
Anaplastic astrocytoma, IDH-mutant	47	43 years	24–65 years	3765 ± 282/mm^2^	162 ± 17 mm^2^	0.74	frontal
Anaplastic astrocytoma, IDH-wildtype	47	54 years	12–79 years	4633 ± 361/mm^2^	85 ± 14 mm^2^	0.57	temporal
Anaplastic ependymoma	50	7 years	0.3–77 years	5571 ± 298/mm^2^	138 ± 16 mm^2^	0.92	fourth ventricle
Anaplastic ganglioglioma	5	36 years	14–70 years	3129 ± 801/mm^2^	96 ± 44 mm^2^	NA	temporal
Anaplastic meningioma	46	63 years	30–84 years	6291 ± 380/mm^2^	223 ± 17 mm^2^	1.19	frontal
Anaplastic oligodendroglioma, IDH-mutant and 1p/19q codeleted	91	49 years	30–78 years	4899 ± 217/mm^2^	162 ± 11 mm^2^	0.72	frontal
Anaplastic pleomorphic xanthoastrocytoma	1	48 years	NA	2933/mm^2^	245 mm^2^	NA	cerebral, NOS
Angiocentric glioma	3	8 years	3–35 years	3519/mm^2^	138 mm^2^	2	temporal
Angiomatous meningioma	32	60 years	43–79 years	4185 ± 330/mm^2^	201 ± 14 mm^2^	1.13	frontal
Angiosarcoma	2	66 years	53–79 years	2440/mm^2^	112 mm^2^	NA	cerebral, NOS
Astroblastoma	7	40 years	12–83 years	5417 ± 440/mm^2^	120 ± 24 mm^2^	1.33	frontal
Atypical choroid plexus papilloma	4	26 years	0.3–56 years	6555/mm^2^	98 mm^2^	1	lateral ventricle
Atypical meningioma	83	58 years	13–92 years	6901 ± 222/mm^2^	225 ± 11 mm^2^	1.13	frontal
Atypical teratoid/rhabdoid tumour	17	3 years	0.3–10 years	6846 ± 791/mm^2^	61 ± 21 mm^2^	1.29	temporal
CNS ganglioneuroblastoma	1	0 years	NA	7247/mm^2^	306 mm^2^	NA	frontal
Cellular schwannoma	25	54 years	27–79 years	7459 ± 526/mm^2^	128 ± 20 mm^2^	1.08	spinal
Central neurocytoma	20	28 years	6–41 years	8053 ± 650/mm^2^	110 ± 22 mm^2^	0.67	lateral ventricle
Cerebellar liponeurocytoma	4	50 years	43–57 years	6924/mm^2^	125 mm^2^	NA	cranial nerves
Chondrosarcoma	21	37 years	6–73 years	5642 ± 621/mm^2^	132 ± 28 mm^2^	1.33	cranial, NOS
Chordoid glioma of the third ventricle	4	34 years	26–42 years	5074/mm^2^	36 mm^2^	0.33	third ventricle
Chordoid meningioma	12	47 years	35–73 years	4408 ± 776/mm^2^	210 ± 17 mm^2^	3	cranial, NOS
Chordoma	28	61 years	4–85 years	2808 ± 342/mm^2^	114 ± 16 mm^2^	0.56	cranial, NOS
Choriocarcinoma	1	76 years	NA	8954/mm^2^	131 mm^2^	NA	sellar region
Choroid plexus carcinoma	7	3 years	0.5–46 years	6778 ± 691/mm^2^	100 ± 35 mm^2^	NA	cerebral, NOS
Choroid plexus papilloma	21	29 years	0.2–78 years	5454 ± 467/mm^2^	156 ± 26 mm^2^	0.91	fourth ventricle
Clear cell meningioma	13	39 years	8–74 years	5027 ± 648/mm^2^	182 ± 32 mm^2^	1.17	sellar region
Crystal-storing histiocytosis	1	62 years	NA	1435/mm^2^	211 mm^2^	NA	NA
Desmoplastic infantile astrocytoma and ganglioglioma	11	1 years	0.5–23 years	5206 ± 642/mm^2^	180 ± 37 mm^2^	1.5	parietal
Diffuse astrocytoma, IDH-mutant	70	37 years	18–60 years	3013 ± 171/mm^2^	105 ± 12 mm^2^	0.64	frontal
Diffuse astrocytoma, IDH-wildtype	19	58 years	20–77 years	2730 ± 315/mm^2^	90 ± 23 mm^2^	0.36	frontal
Diffuse large B-cell lymphoma of the CNS	59	68 years	9–84 years	6021 ± 450/mm^2^	90 ± 13 mm^2^	1.46	frontal
Diffuse leptomeningeal glioneuronal tumour	1	2 years	NA	8070/mm^2^	8 mm^2^	NA	frontal
Diffuse midline glioma, H3 K27M-mutant	21	19 years	3–64 years	4258 ± 460/mm^2^	26 ± 7 mm^2^	1.1	brain stem
Dysembryoplastic neuroepithelial tumour	25	31 years	8–57 years	2410 ± 196/mm^2^	76 ± 16 mm^2^	0.79	temporal
Dysplastic cerebellar gangliocytoma	1	38 years	NA	2345/mm^2^	196 mm^2^	NA	cerebellar
EBV-positive diffuse large B-cell lymphoma, NOS	1	34 years	NA	6595/mm^2^	5 mm^2^	NA	frontal
Embryonal carcinoma	1	39 years	NA	4888/mm^2^	291 mm^2^	NA	parietal
Embryonal tumour with multilayered rosettes, C19MC-altered	3	2 years	2–3 years	6087/mm^2^	231 mm^2^	2	parietal
Ependymoma	46	49 years	2–78 years	4813 ± 347/mm^2^	94 ± 12 mm^2^	0.96	spinal
Ependymoma, RELA fusion-positive	6	12 years	4–55 years	5814 ± 1401/mm^2^	138 ± 40 mm^2^	0.5	lateral ventricle
Epitheloid MPNST	1	50 years	NA	3003/mm^2^	70 mm^2^	NA	other
Erdheim-Chester disease	1	57 years	NA	2194/mm^2^	239 mm^2^	NA	NA
Ewing sarcoma	4	6 years	0.8–28 years	8370/mm^2^	91 mm^2^	1	spinal
Extraventricular neurocytoma	1	36 years	NA	1193/mm^2^	107 mm^2^	NA	spinal
Fibrosarcoma	2	27 years	20–34 years	4639/mm^2^	199 mm^2^	1	cerebral, NOS
Fibrous meningioma	57	58 years	12–84 years	6103 ± 237/mm^2^	228 ± 13 mm^2^	6.12	cranial, NOS
Follicular lymphoma	3	62 years	62–64 years	8741/mm^2^	276 mm^2^	2	occipital
Gangliocytoma	1	36 years	NA	1127/mm^2^	10 mm^2^	NA	occipital
Ganglioglioma	88	21 years	2–65 years	2932 ± 153/mm^2^	110 ± 9 mm^2^	0.6	temporal
Ganglioneuroma	2	33 years	27–39 years	4228/mm^2^	212 mm^2^	1	other
Gemistocytic astrocytoma, IDH-mutant	6	38 years	29–56 years	2036 ± 228/mm^2^	121 ± 25 mm^2^	0.5	temporal
Germinoma	20	16 years	9–33 years	7091 ± 686/mm^2^	21 ± 6 mm^2^	0.11	diencephalon
Giant cell glioblastoma	21	43 years	11–86 years	3170 ± 301/mm^2^	181 ± 20 mm^2^	0.62	temporal
Glioblastoma, IDH-mutant	34	38 years	25–73 years	4867 ± 296/mm^2^	172 ± 19 mm^2^	1	frontal
Glioblastoma, IDH-wildtype	474	62 years	17–87 years	4481 ± 96/mm^2^	151 ± 5 mm^2^	0.66	temporal
Gliosarcoma	59	57 years	9–86 years	4794 ± 276/mm^2^	221 ± 14 mm^2^	0.44	temporal
Granular cell tumour of the sellar region	1	46 years	NA	2172/mm^2^	97 mm^2^	NA	sellar region
Haemangioblastoma	88	50 years	16–81 years	5119 ± 185/mm^2^	109 ± 10 mm^2^	1	cerebellar
Haemangioma	30	51 years	0.2–76 years	2796 ± 292/mm^2^	133 ± 15 mm^2^	2	cranial, NOS
Haemangiopericytoma	34	39 years	25–83 years	9064 ± 489/mm^2^	186 ± 18 mm^2^	0.48	cranial, NOS
Hybrid nerve sheath tumours	3	58 years	32–72 years	3342/mm^2^	227 mm^2^	0.5	spinal
Immature teratoma	7	15 years	0.0–56 years	7927 ± 913/mm^2^	107 ± 28 mm^2^	0.4	third ventricle
Immunodeficiency-associated CNS lymphoma	5	53 years	31–73 years	5209 ± 1227/mm^2^	65 ± 34 mm^2^	0.67	cerebral, NOS
Inflammatory myofibroblastic tumour	1	26 years	NA	5226/mm^2^	298 mm^2^	NA	frontal
Intravascular large B-cell lymphoma	2	70 years	62–78 years	1242/mm^2^	427 mm^2^	NA	NA
Juvenile xanthogranuloma	1	23 years	NA	12519/mm^2^	75 mm^2^	NA	NA
Langerhans cell histiocytosis	32	13 years	1–53 years	6848 ± 565/mm^2^	104 ± 19 mm^2^	0.78	parietal
Leiomyoma	1	50 years	NA	1864/mm^2^	28 mm^2^	NA	cranial, NOS
Leiomyosarcoma	4	58 years	50–77 years	6955/mm^2^	213 mm^2^	1	occipital
Lipoma	38	10 years	0.3–76 years	757 ± 66/mm^2^	120 ± 16 mm^2^	0.9	spinal
Liposarcoma	1	52 years	NA	2697/mm^2^	107 mm^2^	NA	spinal
Low-grade B-cell lymphomas of the CNS	13	67 years	50–83 years	8087 ± 1088/mm^2^	86 ± 25 mm^2^	1.17	spinal
Lymphoplasmacyte-rich meningioma	2	46 years	37–55 years	9817/mm^2^	37 mm^2^	1	cranial, NOS
MALT lymphoma of the dura	5	68 years	39–79 years	9843 ± 1672/mm^2^	39 ± 23 mm^2^	1.5	cranial, NOS
Malignant peripheral nerve sheath tumour	15	61 years	17–81 years	5886 ± 794/mm^2^	136 ± 28 mm^2^	0.88	spinal
Mature teratoma	6	10 years	0.2–49 years	3259 ± 760/mm^2^	135 ± 45 mm^2^	0.2	spinal
Medulloblastoma, SHH-activated and TP53-mutant	3	16 years	0.5–52 years	9539/mm^2^	117 mm^2^	2	cerebellar
Medulloblastoma, SHH-activated and TP53-wildtype	9	30 years	1–75 years	10544 ± 581/mm^2^	185 ± 38 mm^2^	0.5	cerebellar
Medulloblastoma, WNT-activated	7	13 years	6–65 years	7641 ± 954/mm^2^	79 ± 20 mm^2^	0.4	fourth ventricle
Medulloblastoma, non-WNT/non-SHH	32	8 years	3–34 years	8799 ± 412/mm^2^	113 ± 13 mm^2^	0.33	fourth ventricle
Melanotic schwannoma	3	64 years	51–69 years	3110/mm^2^	55 mm^2^	0.5	cranial nerves
Meningeal melanocytoma	5	51 years	35–54 years	8152 ± 1898/mm^2^	172 ± 60 mm^2^	0.67	spinal
Meningeal melanoma	2	61 years	51–71 years	6763/mm^2^	160 mm^2^	NA	spinal
Meningothelial meningioma	104	55 years	25–88 years	5951 ± 212/mm^2^	162 ± 10 mm^2^	4.47	cranial, NOS
Metaplastic meningioma	4	75 years	56–85 years	4613/mm^2^	226 mm^2^	NA	frontal
Metastatic tumours	47	58 years	38–78 years	5092 ± 399/mm^2^	159 ± 14 mm^2^	0.88	spinal
Microcystic meningioma	23	48 years	33–75 years	4475 ± 382/mm^2^	213 ± 23 mm^2^	2.83	frontal
Mixed germ cell tumour	5	20 years	12–44 years	4379 ± 1084/mm^2^	142 ± 45 mm^2^	0.25	spinal
Myxopapillary ependymoma	23	35 years	11–71 years	3188 ± 360/mm^2^	154 ± 16 mm^2^	0.64	spinal
Neurofibroma	16	44 years	0.7–65 years	3640 ± 446/mm^2^	151 ± 29 mm^2^	0.6	spinal
Olfactory neuroblastoma	10	58 years	27–69 years	5053 ± 780/mm^2^	213 ± 34 mm^2^	0.25	cranial, NOS
Oligodendroglioma, IDH-mutant and 1p/19q codeleted	85	46 years	12–73 years	3587 ± 174/mm^2^	136 ± 12 mm^2^	0.7	frontal
Osteochondroma	1	14 years	NA	2388/mm^2^	39 mm^2^	NA	spinal
Osteoma	9	48 years	40–69 years	1570 ± 638/mm^2^	107 ± 33 mm^2^	1.25	frontal
Osteosarcoma	8	30 years	17–54 years	4328 ± 498/mm^2^	176 ± 20 mm^2^	3	cerebral, NOS
Papillary craniopharyngioma	13	61 years	44–82 years	4941 ± 427/mm^2^	65 ± 13 mm^2^	0.86	sellar region
Papillary ependymoma	2	35 years	35–35 years	5573/mm^2^	146 mm^2^	NA	spinal
Papillary glioneuronal tumour	2	12 years	12–13 years	5877/mm^2^	117 mm^2^	NA	cerebral, NOS
Papillary meningioma	3	38 years	20–61 years	7270/mm^2^	198 mm^2^	NA	temporal
Papillary tumour of the pineal region	11	17 years	4–48 years	6094 ± 807/mm^2^	82 ± 28 mm^2^	1.75	third ventricle
Paraganglioma	17	54 years	25–69 years	6734 ± 468/mm^2^	165 ± 24 mm^2^	0.89	spinal
Perineurioma	1	23 years	NA	3580/mm^2^	26 mm^2^	NA	other
Pilocytic astrocytoma	173	11 years	0.6–51 years	3327 ± 117/mm^2^	105 ± 8 mm^2^	0.66	cerebellar
Pilomyxoid astrocytoma	24	7 years	0.4–56 years	4073 ± 362/mm^2^	45 ± 11 mm^2^	1	cranial nerves
Pineal parenchymal tumour of intermediate differentiation	6	44 years	9–55 years	9287 ± 619/mm^2^	86 ± 44 mm^2^	0.2	diencephalon
Pineoblastoma	5	23 years	1–67 years	7436 ± 1352/mm^2^	87 ± 43 mm^2^	4	third ventricle
Pineocytoma	6	19 years	1–40 years	4086 ± 1245/mm^2^	60 ± 38 mm^2^	0.5	diencephalon
Pituicytoma	3	47 years	27–67 years	5231/mm^2^	33 mm^2^	2	sellar region
Pituitary adenoma	99	54 years	16–80 years	7842 ± 225/mm^2^	56 ± 6 mm^2^	0.98	sellar region
Pleomorphic xanthoastrocytoma	21	29 years	5–72 years	4215 ± 368/mm^2^	145 ± 23 mm^2^	1.38	temporal
Plexiform neurofibroma	1	12 years	NA	12310/mm^2^	66 mm^2^	NA	NA
Psammomatous meningioma	28	66 years	29–83 years	5201 ± 372/mm^2^	125 ± 15 mm^2^	8.33	spinal
Rhabdoid meningioma	5	63 years	52–89 years	5016 ± 475/mm^2^	235 ± 62 mm^2^	0.67	occipital
Rhabdomyosarcoma	3	51 years	49–62 years	2474/mm^2^	299 mm^2^	2	cranial, NOS
Rosette-forming glioneuronal tumour	11	25 years	13–47 years	3997 ± 822/mm^2^	56 ± 21 mm^2^	1.75	cerebellar
Schwannoma	81	53 years	14–78 years	5715 ± 229/mm^2^	124 ± 10 mm^2^	0.93	cranial nerves
Secretory meningioma	41	58 years	40–80 years	6112 ± 313/mm^2^	154 ± 16 mm^2^	12.67	cranial, NOS
Spindle cell oncocytoma	1	47 years	NA	4958/mm^2^	13 mm^2^	NA	NA
Subependymal giant cell astrocytoma	14	15 years	10–33 years	3336 ± 447/mm^2^	136 ± 31 mm^2^	0.56	lateral ventricle
Subependymoma	24	54 years	8–81 years	1829 ± 151/mm^2^	125 ± 20 mm^2^	1.18	lateral ventricle
T-cell and NK/T-cell lymphomas of the CNS	1	54 years	NA	7400/mm^2^	18 mm^2^	NA	cerebral, NOS
Tanycytic ependymoma	1	41 years	NA	5440/mm^2^	232 mm^2^	NA	spinal
Teratoma with malignant transformation	1	40 years	NA	7202/mm^2^	122 mm^2^	NA	frontal
Transitional meningioma	68	58 years	29–82 years	7221 ± 213/mm^2^	203 ± 13 mm^2^	2.09	frontal
Undifferentiated pleomorphic sarcoma	1	62 years	NA	5773/mm^2^	401 mm^2^	NA	cranial, NOS

Cellularity and Tissue area are given as [mean ± SEM].
